# Aggressive surgery for advanced ovarian cancer performed by a multidisciplinary team: A retrospective analysis on a large series of patients

**DOI:** 10.1016/j.sopen.2019.05.005

**Published:** 2019-06-30

**Authors:** Stefano Rausei, Stefano Uccella, Valentina D'Alessandro, Baldo Gisone, Francesco Frattini, Georgios Lianos, Francesca Rovera, Luigi Boni, Gianlorenzo Dionigi, Fabio Ghezzi

**Affiliations:** a Department of Surgery, ASST Valle Olona, Gallarate (VA), Italy; b Department of Obstetrics and Gynecology, Ospedale degli Infermi, Biella, Italy; c Department of Gynecology, ASST Settelaghi, University of Insubria, Varese, Italy; d Department of Surgery, ASST Settelaghi, University of Insubria, Varese, Italy; e Department of Surgery, Ioannina, Greece; f Department of Surgery, IRCCS Ca' Granda - Policlinico Hospital, University of Milan, Milan, Italy; g Department of Human Pathology in Adulthood and Childhood, University of Messina, Messina, Italy

## Abstract

**Background:**

To evaluate the impact of extended surgical treatment performed by a team of gynecologists and general surgeons on postoperative morbidity and survival of patients with advanced ovarian cancer.

**Methods:**

We collected data of 156 patients with advanced ovarian cancer stage IIb-III-IV according to International Federation of Gynecology and Obstetrics classification and treated with primary cytoreduction. End points were perioperative and postoperative complications and cancer-related survival.

**Results:**

In 51 cases (51/156, 32.7%) a multivisceral resection was completed. Postoperative complications occurred in 52 cases (33.3%). The duration of the surgical procedure as well as the need for diaphragmatic peritonectomy were the factors independently associated with the development of postoperative complications. Five-year cancer-related survival rate was of 50.7%: only histotype and residual tumor resulted significantly associated.

**Conclusions:**

Our results highlight the importance of a team of gynecologists and general surgeons with specific interests and skills to achieve cytoreduction as rapidly as possible, even when it implies very complex maneuvers.

## **INTRODUCTION**

Patients with advanced ovarian cancer (AOC) benefit from aggressive surgical management: actually, robust evidences support the association between microscopic residual disease after surgery and long-term survival [1], [2], [3], [4], [5], [6], [7], [8], [9]. For each 10% cytoreduction increase, a 5.5% increase in median survival time is estimated [10]. However, in a lot of cases of advanced tumors, to perform effective cytoreductive surgery means to extensively treat very diffuse peritoneal carcinomatosis: this surgery includes procedures on multiple viscera and related structures of the upper and lower abdomen [11]. Hence, to increase the probability of optimal cytoreduction, several multidisciplinary approaches have been introduced: the neoadjuvant and adjuvant therapies [12], [13] as well as a multispecialist surgery (beyond the conventional gynecologic oncologists) [14].

This monocentric retrospective study reviews the impact of extended surgical treatment performed by a team of gynecologists and general surgeons with specific interests and skills in cytoreduction on postoperative morbidity and survival of patients with AOC.

## **MATERIALS AND METHODS**

### Study Design

During the study period, we observed 208 patients with AOC, but 52 were excluded from primary debulking surgery: 2 were excluded because of anesthesiological contraindications to aggressive surgery and 50 because we considered that even aggressive surgery would not have allowed us to obtain no residual tumor. These 50 patients were submitted to neoadjuvant chemotherapy and, when possible, subsequent interval debulking surgery. Finally, we retrospectively collected data of 156 patients diagnosed with AOC stage IIb-III-IV according to International Federation of Gynecology and Obstetrics (FIGO) classification (according to the staging system in effect at the beginning of the study [15]) and treated with upfront primary cytoreductive surgery between May 2002 and September 2017 in the Department of Gynecology of University of Insubria. A fully written informed content was obtained from all patients and study data are available in an Institutional Registry according to the declaration of Helsinki. For this retrospective study, an institutional review board approval was obtained from the University of Insubria.

All patients were considered fit for surgery after careful preoperative evaluation by a team of anesthesiologist, gynecologist and general surgeon. General surgeons were S.R., F.F., L.B. and G.D.; gynecologists were S.U. and F.G. Weekly, our multidisciplinary tumor board discussed every case in order to define therapeutic strategy.

In all cases, staging protocol included CT scan of thorax, abdomen and pelvis and an immediately preoperative laparoscopic evaluation to check the feasibility of aggressive surgery. Before performing midline xifo-pubic laparotomy, patients were submitted to diagnostic laparoscopic to assess the likelihood of cytoreduction. After 2010, the Peritoneal Index described by Fagotti [16] with its modifications and the relative cut-offs were used to discriminate patients who were suitable for primary cytoreduction from those who could benefit more from neoadjuvant chemotherapy.

### Surgical Treatment

Once the possibility of a major cytoreductive surgery was ensured, patients were treated with hysterectomy, bilateral adnexectomy, radical omentectomy, and, when needed, pelvic and/or lumboaortic lymphadenectomy and appendectomy by gynecologists. When a diffuse involvement of peritoneal viscera surface was confirmed, resections of gastrointestinal tube (stomach, small bowel, large bowel), gallbladder, liver, and spleen were performed by general surgeons depending on which organs were macroscopically affected by the tumor. In some patients, peritonectomy of the pelvis, abdomen and diaphragm was completed. R0 resection was defined intraoperatively by the surgeons. The restorative gastrointestinal reconstructions were the first surgical option, but in some cases of low rectal resection preoperatively treated by chemotherapy the stoma option was considered.

### Follow-Up

In the majority of the patients, a postoperative CT scan was performed before the initiation of adjuvant chemotherapy and there was no case of inconsistency between postoperative radiological imaging and surgical notes reporting R0 resection.

A careful follow-up by clinical evaluation, seric tumoral markers and CT/MR scan every 3, 6 and/or 12 months was achieved. Last contact for all patients was in June 2018 without any loss to follow-up.

### Statistical Analysis

End points were perioperative and postoperative complications (assessed according to Clavien-Dindo classification [17]) and cancer-related survival (CRS).

Patient- and tumor-related factors considered in the analysis were age, body mass index (BMI), FIGO stage, grading, histotype and presence of ascites.

Surgery-related variables were multivisceral resection, resected organs (stomach, jejunum-ileum, colon, spleen, liver), peritonectomy (pelvis and/or diaphragm), residual disease, stoma, and procedure duration.

Neoadjuvant and adjuvant therapies were also included among factors assessed.

Comparisons between different groups were performed by nonparametric tests (i.e., χ^2^ and Mann Whitney) as appropriate. Multivariate analysis was conducted by logistic backward stepwise regression, with a model including only factors probably associated with end points (*P* < 0.1).

About survival analysis, follow-up data have been considered from the day of surgery to the last contact or the cancer-related death. Patients dead for other causes have been censored at last contact. The survival analysis results were achieved using Kaplan–Meier method with log-rank test. The analysis was performed using SPSS software for Windows.

## **RESULTS**

Among 156 patients treated by cytoreductive surgery, a R0 resection was achieved in 110 patients (70.5%). In 51 cases (51/156, 32.7%) a multivisceral resection was completed: specifically, bowel resections was performed in 50 out of these 51 patients (98.0%) and 38 patients (39/51, 76.4%) received diaphragmatic peritonectomy. In these patients the multivisceral approach allowed to obtain a R0 resection rate comparable to that observed in the other patients (74.5%, 38/51 versus 68.5%, 72/105; *P* = ns). Table 1 reports details of all variables considered in the analysis.

**Table 1:** Details of all variables considered in the analysis

Variable	# Patients	Median (range) or %
*Age (years)*	156	62 (29–86)
*BMI*	156	23.7 (17–38)
*Histotype*		
Serous carcinoma	98	62.8
Non-serous carcinoma	58	37.2
*FIGO Stage*		
II	3	1.9
III	135	86.5
IV	18	11.5
*Grading*		
1–2	23	14.7
3	133	85.2
*Multivisceral resection*		
Yes	51	32.7
No	105	67.3
*No. resected organs*	51	1 (1–4)
*Residual (mm)*		
0	110	70.5
< 10	46	29.4
*Stomach resection*		
Yes	2	1.3
No	154	98.7
*Jejunum-ileum resection*		
Yes	14	9.0
No	142	91.0
*Colon resection*		
Yes	45	28.8
No	111	71.2
*Splenectomy*		
Yes	14	9.0
No	142	91.0
*Liver resection*		
Yes	2	1.3
No	154	98.7
*Pelvic peritonectomy*		
Yes	91	58.3
No	65	41.7
*Diaphragmatic peritonectomy*		
Yes	39	25.0
No	117	75.0
*Pelvic lymphadenectomy*		
Yes	91	58.3
No	65	41.7
*Lumboaortic lymphadenectomy*		
Yes	67	42.9
No	89	57.1
*Presence of ascites*		
Yes	78	50.0
No	78	50.0
*Procedure duration (min)*	156	240 (40–600)
*Stoma*		
Yes	13	8.3
No	143	91.7
*Neoadjuvant chemotherapy*		
Yes	12	7.7
No	144	92.3
*Adjuvant chemotherapy (cycles)*	144	6 (1–49)

Postoperative complications occurred in 52 cases (33.3%): according to the Clavien-Dindo classification a grade I-II was observed in 25.0% of cases (39/156), a grade III in 7.1% (11/156), a grade IV in 0.6% (1/156), similarly to the rate reported for grade V (0.6%, 1/156) (Table 2). Specifically, complications induced a longer postoperative hospital stay (median 11 days vs median 8 in non-complicated patients, *P* < .01).

**Table 2:** Postoperative complications assessed according to Clavien-Dindo classification [16]

Clavien-Dindo classification	Complication	
**Grade I–II (39)**	Wound infection	10 (25.7)
	Ileus	9 (23.1)
	Anemization	7 (17.9)
	Abdominal fluid collection	6 (15.4)
	Other	7 (17.9)
**Grade III (11)**	Bleeding	5 (45.4)
	Bowel stump/anastomosis leak	4 (36.4)
	Ileus	2 (18.2)
**Grade IV (1)**	Bleeding	1 (100)
**Grade V (1)**	Anastomosis leak	1 (100)

Results of univariate and multivariate analysis for complications are shown in Table 3.

**Table 3:** Results of univariate and multivariate analysis for complications; for Constant in logistic regression: *P* = .021, RR = 5.458

Variable	# Complicated patients	%	Univariate analysis *(P)*	Multivariate analysis	
				*P*	RR
*Multivisceral resection*			<.001		
Yes	28/51	54.9			
No	24/105	22.8			
*Jejunum-ileum resection*			.012		
Yes	8/14	57.1			
No	44/142	31.0			
*Colon resection*			<.001		
Yes	25/45	55.6			
No	27/111	24.3			
*Splenectomy*			.005		
Yes	9/14	64.3			
No	43/142	30.3			
*Diaphragmatic peritonectomy*			<.001	.021	
Yes	22/39	56.4			1
No	30/117	25.6			0.353
*Pelvic lymphadenectomy*			.031		
Yes	37/91	40.7			
No	15/65	23.1			
*Procedure duration*			.001	.044	
≥ 240 min	35/72	48.6			1
< 240 min	17/84	20.3			0.428

**Fig. 1:**
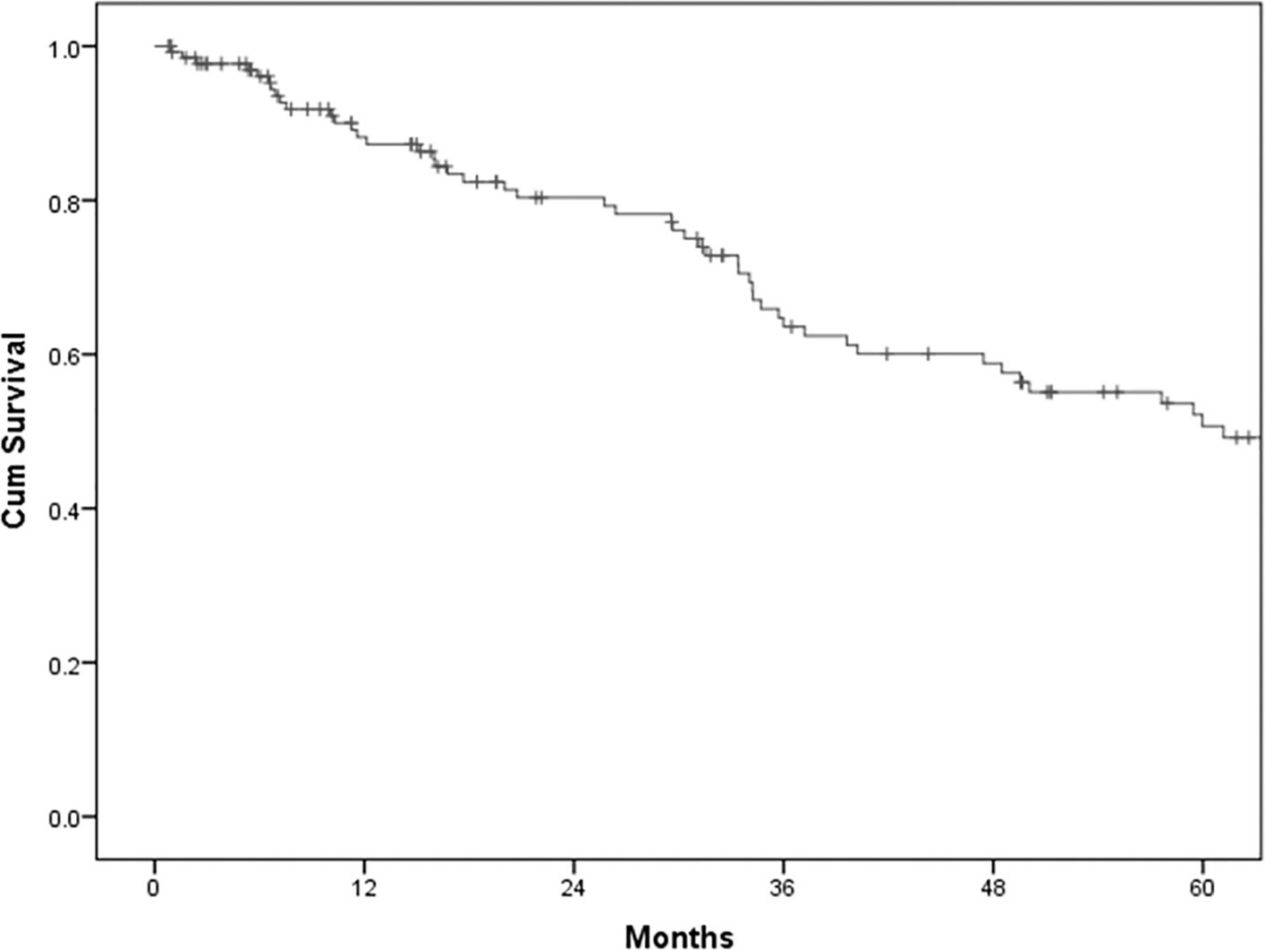
Five-year cancer-related survival.

At a median follow-up of 31.1 months (range 1–189 months), the 5-year CRS rate was of 50.7% (Fig 1).

**Fig. 2:**
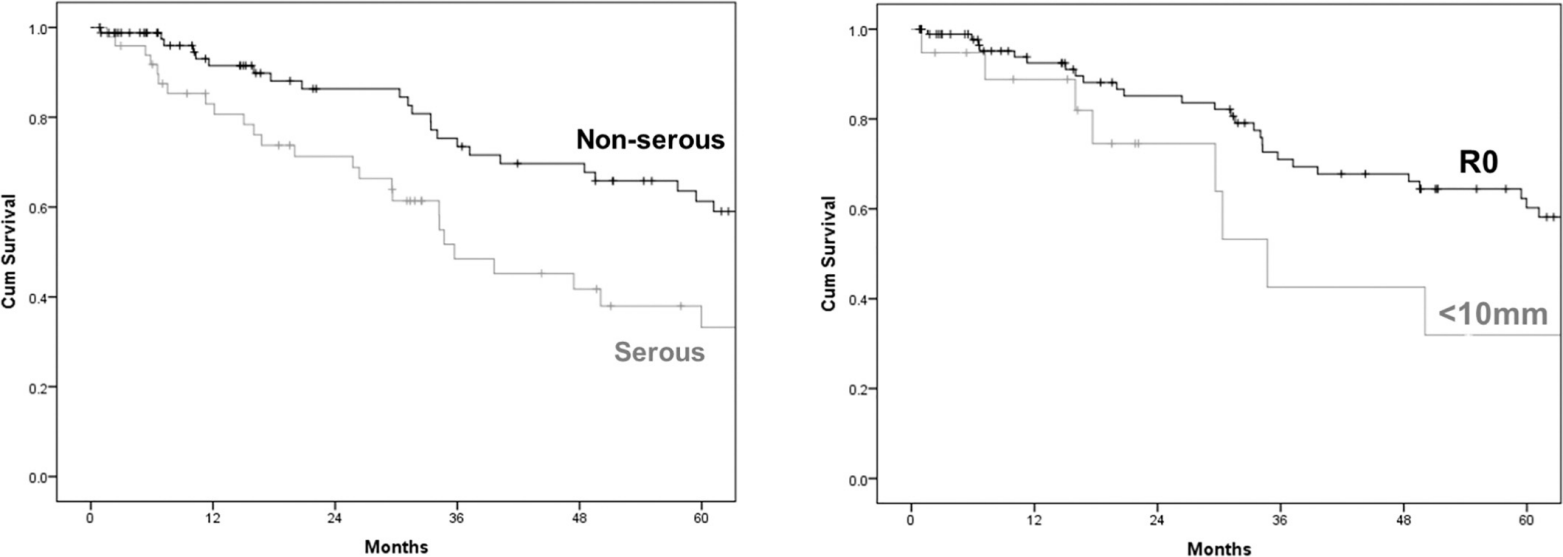
**a-b.** Five-year cancer-related survival according to (a) histotype and (b) residual tumor.

At log-rank test only histotype and residual tumor resulted significantly associated with CRS (Table 4, Fig 2, *A* and *B*).

**Table 4:** Survival analysis by log-rank test

Variable	5 year cancer-related survival (%)	*P*
*Histotype*		
Serous carcinoma	33.2	.002
Non-serous carcinoma	61.3	

*Residual (mm)*		
0	60.2	.002
< 10	31.8

It is to be noted that postoperative complications did not affect survival: complicated patients showed a 5-year CRS of 48.0% versus 51.8% (*P* = ns) observed in patients with a regular postoperative course.

## **DISCUSSION**

Ovarian cancer is usually diagnosed at an advanced stage due to the lack of adequate screening tools and to the paucity of symptoms in early stage disease [18]. Signs and symptoms become manifest only when the tumor mass invades contiguous or far anatomical structures, thus inducing a clinical picture that guides towards the suspicion of a gynecological tumor. Given these premises, over 70% of the patients receive a diagnosis of ovarian cancer only in cases of tumor spread beyond the ovaries (FIGO stages II, III, and IV), with frequent massive involvement of the peritoneal surfaces and intra-abdominal organs. This clinical scenario results in low cure rates and poor prognosis. However, several well-designed studies have shown that a particularly aggressive surgical approach aiming at radically eradicating all the macroscopic localization of advanced ovarian cancer is associated with better survival, in spite of a wide diffusion of the disease [7]. The obvious consequence has been a steep increase in surgical complexity, which necessarily opened the door of gynecological operative theater to general surgeons. Literature recognizes the effective role of a multispecialist surgical team dedicated to AOC cytoreduction [19]: in fact, gynecological surgeons may not always be familiar with multivisceral resection extended, for example, to gastrointestinal organs and diaphragm, making it difficult to know which extensive procedures should be implemented. On the other hand, an aggressive attitude of a general surgeon lacking in knowledge on ovarian cancer therapy might expose to excessive or inadequate treatment.

In our study, we aimed to verify that the cooperation of a team of gynecologic oncologists and general surgeons dedicated to AOC and its surgical aggressive behavior actually leads to an improvement in the prognosis with an acceptable morbidity.

In our series of 51 multivisceral resections (32.7%), the organs involved in the procedures are consistent with Literature data [10]. Also in this analysis the multivisceral resection exposes the patient to a non-negligible risk of developing morbidity (54.9% versus 22.8%); this risk is much higher in patients who, due to the advanced disease only, suffer of poor general condition with low performance status or malnutrition [20], [21]. Among the variables related to multivisceral surgery, logistic regression identified the duration of the surgical procedure as well as the need for diaphragmatic peritonectomy as the factors independently associated with the development of postoperative complications.

Actually, we are surprised that intestinal resection was not a risk factor for complications: given this result, in order to better “correct” the multivariate analysis, we tried to analyze different groups classified according to a lot of factors: bowel (small and large together) resection, contamination, operative severity score, stoma (small and large together, end stoma and loop stoma together). At the end, we had to confirm the first model.

From a practical point of view, our results highlight the importance of a surgical team able to achieve cytoreduction as rapidly as possible, even when it implies very complex maneuvers. In fact, diaphragmatic peritonectomy is a challenging procedure, consisting of peritoneal stripping and electrocoagulation of the peritoneal nodules of carcinomatosis, sometimes up to the level of the suprahepatic and cava veins.

Nevertheless, it is worth to take this risk. Survival of AOC patients benefits from aggressive surgery aiming to R0-resection (Fig 2, *B*) and, opposite to some previous experiences [22], [23], [24], complications did not seem to negatively affect survival in our study population.

This study presents several limitations to be considered when interpreting our results. Firstly, essentially it is an observational study with a retrospective approach. Secondly, multivisceral resections represent only one third of our population and we had no a synchronous arm of patients with the same disease diffusion to compare in terms of outcomes of this aggressive treatment. Finally, we included adjuvant and neoadjuvant chemotherapy in our model, but this variables it was not well detailed.

In conclusion, the struggle between the attempt to guarantee the best possible survival and achieving less complications is very challenging for any surgeon. The aim is providing a good, balanced result: this can be pursued only with a complete collaboration of all different professional figures involved. If general surgeons want to contribute to the cure of AOC, they must know to differentiate carcinomatosis of ovarian cancer and its surgical “possibilities” from their conventional practice in gastrointestinal surgery. In fact, if in gastrointestinal surgical oncology an aggressive approach is not always satisfactory, in AOC it represents a real chance of cure.

## **DISCLOSURES**

**Author Contribution**. Stefano Rausei and Stefano Uccella conceived of the presented study, performed the computations and verified the analytical methods; Valentina D'Alessandro, Baldo Gisone, Francesco Frattini, and Georgios Lianos verified the analytical methods and collected data; Francesca Rovera, Luigi Boni, Gianlorenzo Dionigi, and Fabio Ghezzi supervised the findings of this work. All authors discussed the results and contributed to the final manuscript.

**Conflict of interest**. Authors have no conflict of interest to declare.

**Funding sources**. Authors have no funding sources to declare.
